# Financing Essential HIV Services: A New Economic Agenda

**DOI:** 10.1371/journal.pmed.1001567

**Published:** 2013-12-17

**Authors:** Anna Vassall, Michelle Remme, Charlotte Watts, Timothy Hallett, Mariana Siapka, Peter Vickerman, Fern Terris-Prestholt, Markus Haacker, Lori Heise, Andy Haines, Rifat Atun, Peter Piot

**Affiliations:** 1 SAME Modelling and Economics, Department of Global Health and Development, London School of Hygiene & Tropical Medicine, London, United Kingdom; 2 Imperial College, London, United Kingdom; 3 Faculty of Public Health and Policy, London School of Hygiene & Tropical Medicine, London, United Kingdom; 4 Harvard School of Public Health, Harvard University, Boston, Massachusetts, United States of America; 5 London School of Hygiene & Tropical Medicine, London, United Kingdom

## Abstract

Anna Vassall and colleagues discuss the need for, and challenges facing, innovative and sustainable financing of the HIV response.

*Please see later in the article for the Editors' Summary*

**Summary Points**

- The financing needs of the HIV response will remain substantial for many years to come, with current commitments becoming increasingly out of line with future fiscal liabilities.
- A change in economic approach will be required, drawing on increased domestic funding, improvements in efficiency, and identification of innovative new funding streams.
- Organisations providing HIV services must critically examine and justify their costs and priorities, become increasingly involved in broader health systems strengthening, and find ways to simultaneously support good governance and wider development objectives.
- There is need for a renewed economic case to now be made for a reinvigorated response and a sustainable, long-term national and global financial commitment to ending AIDS.

## The Need for Innovative Sustainable Financing of the HIV Response

Despite increasing optimism, the end of AIDS is not in sight. Whereas in 1990, HIV/AIDS ranked 24th out of the top 25 causes of years of life lost globally, in 2010 it ranked sixth [1]. In 2011, an estimated 1.7 million people died of AIDS-related causes and 2.5 million were newly infected with the virus; and the number of people living with HIV continues to outgrow the financial and human resources currently allocated to treat them [2]. While the need for HIV services increases, health system constraints limit many countries' ability to meet targets for scaling up core HIV services [2]. Considerable demand-side barriers remain, as evidenced by the limited uptake of male circumcision [3]. Structural factors, including poverty, stigma, and gender inequality continue to underpin HIV vulnerability [4].

Sustainable financing is essential for an expanded HIV response. UNAIDS has recently estimated that the cost of achieving universal access to HIV prevention, treatment, care, and support in 2015 will be US$22 billion [5]. Future treatment costs remain unaddressed with short-term funding cycles. There is a moral obligation to maintain treatment for those who need it, and considerable resources have been implicitly pre-committed to lifelong HIV treatment and care. In countries such as Swaziland and Uganda, the fiscal liability the commitment to lifelong treatment creates is substantial—and for the next two decades may be up to three times annual gross domestic product (GDP) [6].

## Options and Opportunities

### Domestic Revenues

In most low- and middle-income countries, domestic financing is pivotal to funding the HIV response. While external financing accounts for two-thirds of HIV investment in sub-Saharan Africa, more than two-thirds of general health expenditure is financed from domestic sources, funding the health systems upon which HIV services rely [7]. However, the amount of revenue governments can raise from taxes is constrained due to the risk of excessive taxation dampening nascent economic growth. It can therefore be challenging to identify additional domestic budgetary flexibility for investments in HIV, without compromising fiscal stability [8].

**Figure 1:**
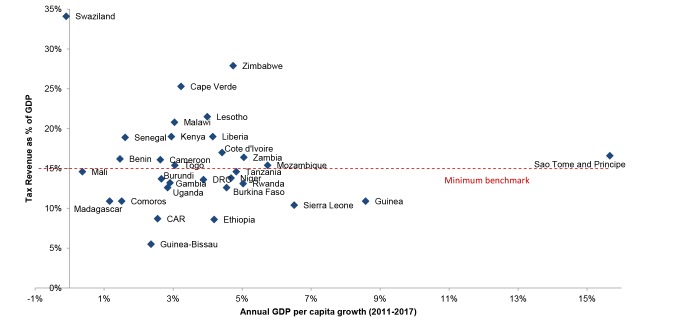
Growth and taxation rates in low- and lower middle-income countries. Governments' capacity to generate revenue is higher than reflected in tax revenue estimates, particularly in oil-producing countries that directly collect profits from oil production. However, most of these countries had data on tax revenue expressed in percent of non-oil GDP only and were therefore excluded for comparability (e.g., Chad, Republic of Congo, Gabon, Ghana, Mauritania). The remaining countries where oil was a source of revenue were Cameroon and Cote d'Ivoire, although for the latter, most of the oil revenue was through taxes (except a small contribution from oil company dividends). Sources: International Monetary Fund World Economic Outlook Database and Country Reports [32]. Tax revenues are from the latest year available between the five-year period from 2007 to 2011. For Uganda and Madagascar, this meant using the IMF projected estimates for 2007, rather than the actual value, while for Kenya, it meant using the IMF estimate for 2009–2010. Average annual GDP per capita growth rates (2011–2017) are authors' calculations from IMF GDP per capita estimates (in constant prices national currency).

Nevertheless, there is potential from the positive economic growth and rising domestic tax revenues forecast in a number of sub-Saharan African countries [9]. For example, Zambia's 5% projected per capita annual GDP growth rate between 2011 and 2017 may generate up to an additional US$21.8 per capita in health expenditures, based on Zambia's current per capita domestic health expenditure of US$63.48 [10]. Of course, such gains will not be possible for all—for example Swaziland's −0.1% projected annual GDP growth rate over the same period offers limited potential for increased health spending. But, even in those countries with slow economic growth, there may be room to increase tax revenues, with many countries still being well below the minimum International Monetary Fund (IMF) benchmark level of 15% of GDP for low-income countries (see Figure 1) [11]. External borrowing can also generate revenue, particularly where there are economic returns to HIV programmes [12]. However, for many low-income countries with high debt-to-GDP ratios, the room to absorb additional lending remains limited [8].

### External Financing

Economic constraints in high-income countries appear to be resulting in a flat-lining of development assistance to health (DAH) [13]. Non-health development priorities are dominating the post-2015 agenda for sustainable development, with only one out of 11 thematic groups addressing health [14]. Moreover, the value of providing development assistance to middle-income countries is being challenged, leaving some high-prevalence countries, such as South Africa, with the prospect of funding much of their own HIV response [15].

Much current attention is being placed on achieving “value for money.” To avoid resource waste through duplication and fragmentation [16], DAH needs to be predictable, aligned to national priorities, and nationally “owned,” as embodied in the Paris Declaration on Aid Effectiveness. The financing for HIV may be better sustained, planned for, and absorbed if it becomes part of the broader shift towards investing in the shared responsibility for universal coverage of essential health services [16]. A long term approach, fully embedded in national expenditure frameworks, may also reduce any substitution effect, whereby external financing replaces domestic financing for HIV [17].

**Figure 2:**
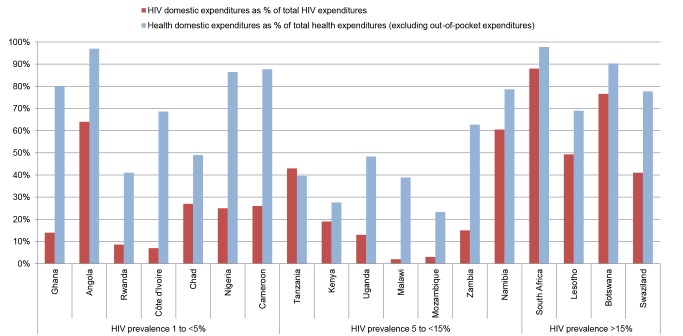
Share of domestic financing in HIV and health expenditures. Countries with the largest numbers of people living with HIV (UNAIDS 2011 estimates) were selected, up until the point where all countries with double-digit HIV prevalence rates were included. Sources: WHO's Global Health Expenditure Database [10] and UNAIDS AIDSinfo database [33]. Countries are ordered from lowest to highest adult HIV prevalence (2011 estimates). HIV spending is from the latest year available (2005–2011), while health expenditure data are 2011 estimates.

Tellingly, the United States President's Emergency Plan for AIDS Relief (PEPFAR) and the Global Fund to Fight AIDS, Tuberculosis and Malaria (GFATM) are now emphasising a transition to “country-led” responses with greater contributions from domestic sources. PEPFAR now requires “cost-sharing assurances” of 25% from governments, while the GFATM requires between 5% and 60% counterpart financing, depending on a country's income classification [18],[19]. These requirements represent a significant change for countries like Malawi—where 98% of HIV spending comes from external sources—and care has to be taken not to reduce domestic funding for other essential health services (Figure 2) as a consequence.

### Innovative Financing Sources

Innovative revenue streams are currently being explored in several countries. These schemes can generate significant funds, as seen in Zimbabwe with the 3% AIDS levy deducted from businesses and formal sector workers' salaries since 2000 [7]. Other options, such as increased “sin taxes” on alcohol, could generate a “double dividend” by simultaneously increasing revenues and decreasing HIV-related risk behaviours [20]. The development of social health insurance could help attract further household resources and may be an important new source of financing in middle-income countries as development assistance scales down [16]. However, none of these options is without challenges. Earmarking restricts the ability of governments to allocate funds efficiently across sectors, and may create a disincentive to use broader tax revenues to fund the HIV response. Health insurance presents considerable challenges in terms of ensuring sustainability, equity, and financing the care of chronic illness. Each of these options therefore needs to be carefully evaluated within the broader framework of health financing reform and on a country-by-country basis.

In addition, between 1990 and 2010, innovative approaches for raising external financing for health in low- and middle-income countries totalled US$6.3 billion, including solidarity levies from global taxes (US$970 million, of which US$580 million was from an airline ticket tax) and funding from novel financing instruments such as Innovative Financing for Immunisation (US$3.7 billion) [21]. Developing these approaches and other sources and the platforms that support them (GAVI, GFATM, UNITAID, etc.) may offer further opportunities for greater and more efficient financing from external sources in the future [22].

### Prioritising Health and HIV

**Figure 3:**
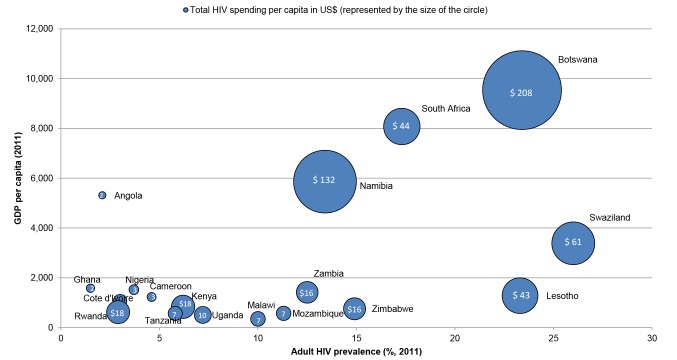
Per capita HIV spending in relation to wealth and disease burden. Countries with the largest numbers of people living with HIV (UNAIDS 2011 estimates) were selected, up until the point where all countries with double-digit HIV prevalence rates were included. Sources: World Bank's World Development Indicators Database [34] and the UNAIDS AIDSinfo database [33]. Adult HIV prevalence and GDP per capita are 2011 estimates, while HIV per capita spending is from the latest year available (2005–2011), but it is unclear from AIDSinfo which currency year is used.

Financing the HIV response must also be achieved without damaging investments in health systems more broadly and other development sectors that are essential for social welfare (in turn addressing a number of the barriers to scaling up the HIV response) [5]. An HIV programme may have important external benefits for sexual and reproductive health; maternal and child health; or provide the necessary health system platforms for managing chronic conditions [23]. Similarly, investments in strengthening health systems or addressing related co-morbidities that compound HIV vulnerability or worsen treatment outcomes are critically important to individuals living with HIV. Although core HIV interventions have been demonstrated to be cost-effective, total HIV spending in sub-Saharan African countries was an estimated 19.4% of total health spending in 2007 (range: 0.7%–64.4%). This amount exceeds the relative burden of HIV disability-adjusted life years (DALYs) [24] and is at least partly due to the relatively high costs of HIV treatment compared to treatment for other prevalent diseases. There remains a difference between the amount spent on the HIV response across countries with a similar GDP per capita and HIV prevalence (Figure 3), and more work is required to understand the optimal level of domestic resourcing for HIV, given competing health sector priorities.

**Figure 4:**
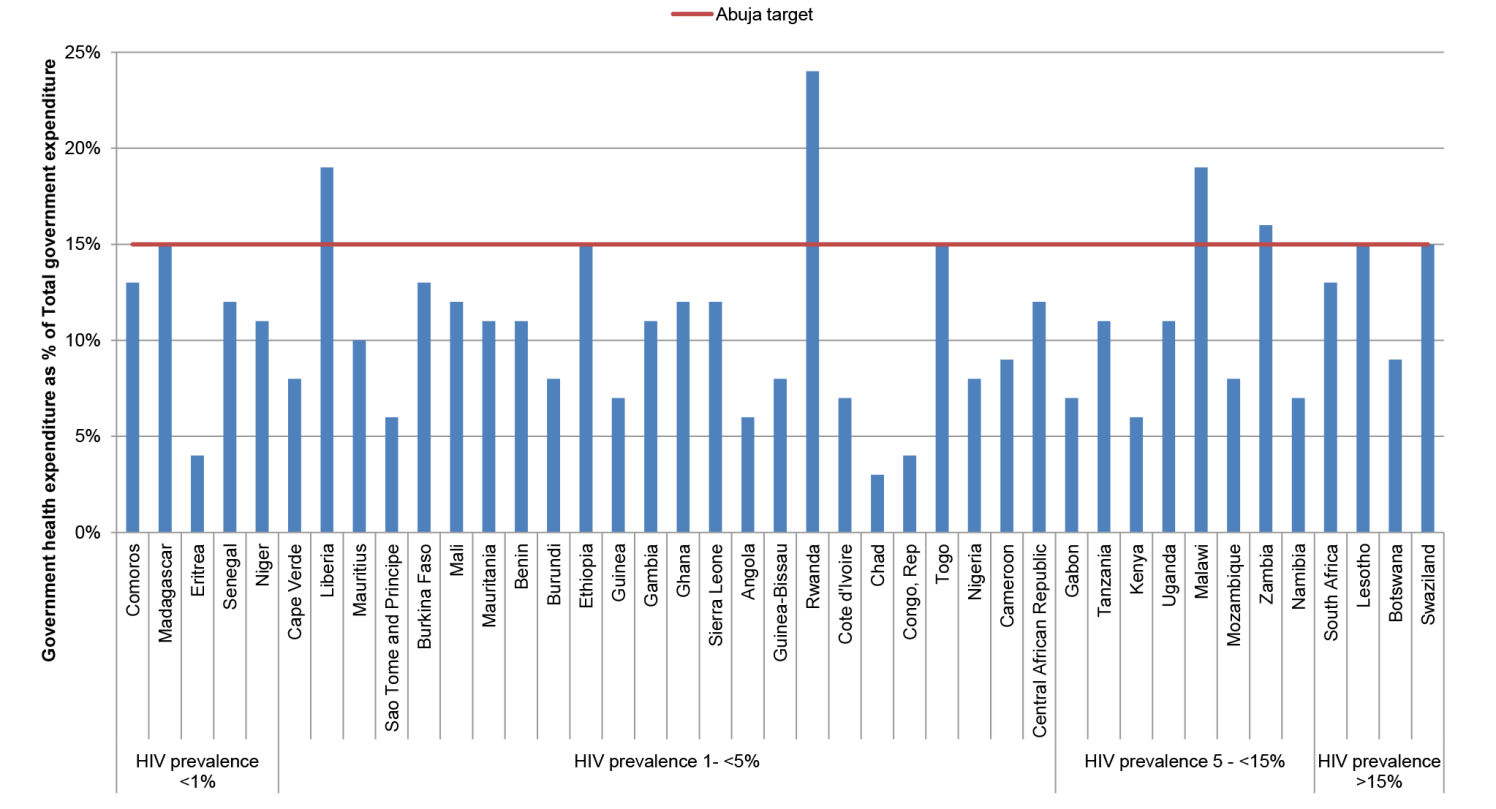
Prioritisation of health in government expenditures. Sources: WHO's Global Health Expenditure Database (2011 estimates for health) [10]. Countries are ordered from lowest to highest adult HIV prevalence (UNAIDS 2011 estimates).

However, few countries have reached the Abuja target of a 15% allocation of the government budget to health (Figure 4) [16], suggesting that for countries like Kenya or Mozambique, where the health sector receives less than 8% of the national budget, organisations working in HIV need to work with others to argue for a re-prioritisation of health more broadly. Ministries of finance (and donors) can be reluctant to allocate more resources to the health sector, when funds remain unspent. Even with political will, limited capacity to effectively spend funds in the health sector can lead to chronic under-spending [25], further illustrating the importance of ensuring that key bottlenecks in broader health systems are fully addressed.

Unfortunately, earmarked external HIV funds have tended to create a sectoral “silo” mentality, which has led HIV programmes to miss out on the many potential synergies within health and with other development sectors. It is now critical to take advantage of the wider benefits generated by some HIV investments and adopt new co-financing approaches between HIV and broader development programmes. For example, programmes that improve livelihood opportunities or help keep young girls in school may yield HIV as well as development benefits [20]. However, at present, the sector-specific perspectives that dominate priority-setting are not designed to recognise and harness such opportunities [26]. Interventions with direct HIV outcomes may, for example, appear more attractive than interventions that could have more structural and long-term benefits for the health or government system as a whole [27]. Approaches through which various sectors pool funds or engage in joint budgeting to fund interventions with multi-sectoral benefits can be found in high-income countries [28], and are worth exploring.

### Efficiency Gains within HIV Programmes

Advocacy pleas for continued funding for the HIV response will also no longer be sufficient without parallel demands for increased efficiency in their use [5]. Improving efficiency is about preventing more new infections and saving more lives by doing the right things for the right populations, as well as delivering quality services at the lowest cost. The World Health Organisation (WHO) estimates that 20%–40% of all resources spent on health are wasted through leakages, inefficient combinations of interventions, and sub-optimal use of medicines and human resources [16]. Good governance is a pre-requisite given that sustaining financing from both domestic and external financing sources is hard to justify when a substantial leakage of funds exists [29]. Blueprint HIV programmes may have resulted in inefficiencies in resource allocation between different HIV interventions, and left some highly vulnerable groups under-served [30]. Increased attention needs to be placed on prioritising those interventions with proven effectiveness resulting in the best patient and population-level outcomes within the resources available. While there has been success in recent years in improving the efficiency of HIV service providers [2], programme costs still require careful scrutiny. Reducing the distortions and duplication inherent in parallel management systems [27]—originally necessary to initiate the rapid scale-up of HIV services—could help redirect scarce resources towards direct service delivery. Improved organisational structures (for example providing integrated service platforms) and community-based delivery may also reduce costs to the health system and patients [5],[31], but these gains have yet to be demonstrated at scale.

## Future Policy and Research Agenda

Adjusting to this new and complex reality will require a concerted global effort to support countries to fully institutionalise their response within domestic governance structures, and move beyond a silo HIV approach. Domestic finance ministries, who will increasingly be at the front-line of the fight against HIV, will need to be supported with new economic, epidemiological, and developmental evidence on how to harness each of these potential areas for increased financing in a way that reflects their national contexts.

This effort will not be without major technical, political, and economic challenges. The magnitude of the conflict between current commitment levels and long term fiscal liabilities is substantial. Increasing domestic financing, improving efficiency, and adopting a bold and innovative financing framework will be central to success. However, none of these options are easy for governments to implement. Increasing domestic financing, particularly in low-income countries, will require creative solutions to ensure the poor are not negatively affected. In some middle-income countries, efforts to increase domestic financing will require a paradigm shift in the HIV community; moving away from highlighting resource needs, towards participating in national planning processes and offering solutions that resonate with those working in broader development policy.

Efforts to improve efficiency will require impartiality to examine trade-offs from spending in one area of HIV response compared to others, some of which may be unpalatable to different domestic interest groups. HIV programmes will need to conduct an open, critical examination of their relative costs compared to other development programmes. In order for donors to champion additional external financing, collaboration, rather than competition, with other development orientated interest groups will be required. The relationship between donors and national governments will need to be carefully navigated and may become increasingly complex as governance becomes central to any offer of co-financing. Finally, the same level of innovation that has produced some of the best HIV technologies will now be required to fund and sustain their use.

To address these challenges there is now an accentuated need for more and better evidence that speaks to the needs of policymakers at the country level. Key evidence gaps include: better understanding how the HIV response can interact with the broader health system and other development sectors; the accurate estimation of the magnitude of the current and future fiscal commitments and financing gaps; and evaluating opportunities for optimising efficiency in service delivery and new financing modalities. The global economic crisis and the hope of ending AIDS do not justify a reduction in our efforts. Rather, there is need for a renewed economic case to now be made—alongside the moral one—for a reinvigorated response and a sustainable, long-term national and global commitment to ending AIDS.
